# COL4A1 Promotes Gastric Cancer Progression by Regulating Tumor Invasion, Tumor Microenvironment and Drug Sensitivity

**DOI:** 10.2174/0109298673351943250314074632

**Published:** 2025-04-07

**Authors:** Xiaojun Qian, Wei Jia, Yuntian Li, Jian Chen, Jinguo Zhang, Yubei Sun

**Affiliations:** 1 Division of Life Sciences and Medicine, Department of Medical Oncology, The First Affiliated Hospital of USTC, University of Science and Technology of China, Hefei, 230001, P.R. China;; 2 Division of Life Sciences and Medicine, Department of Radiation Oncology, The First Affiliated Hospital of USTC, University of Science and Technology of China, Hefei, 230001, P.R. China

**Keywords:** Collagen IV, COL4A1, gastric cancer, immune infiltration, nomogram, western blot

## Abstract

**Background:**

Collagen type IV alpha 1 chain (COL4A1), which has been proven to be a potential biomarker in Gastric Cancer (GC), but its role in tumors and the tumor microenvironment (TME) needs further explanation.

**Methods:**

We analysed the relationship between COL4A1 and clinical characteristics based on The Cancer Genome Atlas (TCGA) database and verified by tissue microarrays as well as GC cell lines using immunohistochemistry, Q-PCR, Western blot, cell proliferation assays, colony formation assays, cell invasion and migration assays. The immune infiltration and drug sensitivity information between high and low COL4A1 expression were analysed by R package and pRRophetic package. Finally, we established a nomogram based on COL4A1 expression using the bootstrap method.

**Results:**

COL4A1 was overexpressed in gastric carcinoma compared with normal gastric tissue, indicating a poor prognosis of GC patients in the TCGA database which were also validated by GC tissue microarrays. GO, KEGG and hallmark enrichment analyses indicated that COL4A1 was mainly associated with the extracellular matrix than malignant proliferation. By siRNA transfection, we found that COL4A1 knockdown inhibited cell colony formation, invasion and migration but did not affect cell proliferation, similar to previous results. Immune infiltration and drug sensitivity analysis showed that COL4A1 was negatively correlated with antitumor immunity and positively correlated with multidrug resistance. By developing a nomogram model based on 8 risk factors, including COL4A1, patients with better clinical outcomes could be accurately distinguished.

**Conclusion:**

COL4A1 is identified as a prognostic marker and potential therapeutic target in gastric cancer. Its overexpression correlates with poor prognosis, tumor invasion, and immunosuppression. A nomogram based on COL4A1 can predict patient outcomes. Future research should validate these findings and explore targeted therapies.

## 1 INTRODUCTION

Patients with unresectable and metastatic gastric cancer (GC) generally require systemic therapy. In addition to chemotherapy [1] and HER2-targeted therapy [2], new agents such as immunotherapy [3, 4] and antiangiogenic therapy [5, 6] are gradually emerging. The Cancer Genome Atlas (TCGA) project performed a comprehensive molecular evaluation of 295 primary gastric adenocarcinomas. It categorized GC into 4 subtypes: EBV-positive tumors, MSI-H tumors, genomically stable tumors, and chromosomal instability (CIN) tumors [7]. However, this classification is limited in clinical application due to the lack of therapeutic guidance, albeit immunotherapy is exclusively used in MSI-H tumors. Clinicians urge to implement biomarkers that directly help individualized treatment strategies, including MSI high (MSI-H), PD-L1, tumor mutation burden (TMB), Epstein‒Barr virus (EBV), and HER2 [8]. In the era of immunotherapy, patients benefited only slightly from immunotherapy, with median overall survival only increasing by about 2 months [9, 10]. More importantly, the incidence of ≥grade 3 adverse events of anti-PD-1/ PD-L1 leading to treatment interruption or even death has become a major concern for clinicians [11-13]. Promising biomarker identification is the simplest and most effective approach to predict and improve the outcomes of GC patients [14, 15]. In addition to trastuzumab targeting HER-2 [2], several biomarkers and corresponding targeted therapies have been investigated, such as AZD4547 targeting FGFR2 [16], AMG337 targeting MET [17], ramucirumab targeting VEGFR2 [5], olaparib targeting PARP [18, 19], strategies targeting inflammatory TME [20] and zolbetuximab targeting Clauding 18.2 (CLDN18.2) [21]. The most promising at this point in time is CLDN18.2, which participates in the proliferation, differentiation, and migration of tumor cells implicated in a number of malignancies, including GC [22]. Various therapeutic approaches have been established based on CLDN18.2, such as monoclonal antibodies (mAbs), bispecific antibodies (BsAbs), chimeric antigen receptor T (CAR-T) cells, and Antibody-Drug Conjugates (ADCs), among which redirected CAR T cells showed promising efficacy against GC in preclinical studies [23].

Collagen type IV alpha 1 chain (CLO4A1) was reported to be associated with poorer overall survival due to its strong correlation with T stage and recurrence of GC based on the GEO database [24]. When identified in GC cell lines, downregulation of COL4A1 expression by shRNA significantly inhibited cell viability, migration, invasion, and EMT of GC cells [25, 26]. It seems that COL4A1 might be a promising therapeutic target. However, more data are needed to determine the importance of COL4A1 in GC cells, especially in TMB, as well as *in vivo* validation using tumor tissues.

In this study, we validated the malignant potential of COL4A1 derived from the TCGA database in GC tissue microarrays and analysed the involved signaling pathways by GO, KEGG and hallmark analysis. The signaling pathways and biological function of COL4A1 were also investigated in gastric cell lines. The immune microenvironment and drug sensitivity were analysed further to assess the potential therapeutic value of this target, and a nomogram based on COL4A1 was established to assess the prognostic value.

## 2 MATERIALS AND METHODS

### 2.1 Cell Culture

GC cells MKN-45 and BGC-823 were obtained from Procell Life Science & Technology Co., Ltd. (Wuhan, China). Cells were cultured in RPMI-1640 medium (Gibco, Gaithersburg, USA) supplemented with 10% fetal bovine serum (BBI Life Sciences Corporation, Shanghai, China). Cells were incubated at 37°C in a humidified atmosphere containing 5% CO_2_.

### 2.2 Real-time Quantitative Pcr

Total RNA from MKN-45 cell lines was extracted by TRIzol™ Reagent (Invitrogen, Carlsbad, CA). Reverse transcription of complementary DNA was obtained using the Superscript Vilo cDNA synthesis kit (Thermo Fisher, Waltham, MA, USA). Real-time quantitative PCR assays were performed using the Bio-Rad CXF96 Sequence Detection System (Applied Biosystems) with SYBR premix Ex Taq (Takara, Dalian, China). The thermal profile consisted of one cycle at 95°C for 60 s, 45 cycles at 95°C for 5 s, and 60°C for 20 s. Data were analysed using the comparative Ct method [27]. The following primers were used (F: forward; R: reverse): COL4A1 F: 5′- CCTCCAGGATTTACCGGACC -3′, R: 5′- TTTCTCCCTTGGTGGCGAAG -3′, GAPDH F: 5′- GTCAAGGCTGAGAACGGGAA, R: 5′-AAATGAGCCCCAGCCTTCTC -3′.

### 2.3 Western Blot Analysis

Cells were collected and washed with PBS 2 times before whole-cell lysates were obtained using SDS sample buffer. Cell lysates were boiled at 100°C for 10 min, subjected to sodium dodecyl sulfate-polyacrylamide gel electrophoresis at the indicated concentration, and then transferred to PVDF membranes (Millipore, Darmstadt, Germany). Five percent milk in PBST containing 0.05% Tween 20 was used to block the membranes. The PVDF membrane was incubated with various primary antibodies at 4° overnight and then incubated with HRP-conjugated secondary antibodies for at least 2 h at room temperature. Images were detected by enhanced chemiluminescence reagent (Thermo Fisher Scientific Inc.) and visualized by a gel imaging analysis system (Bioshine, Shanghai, China). The following antibodies were used: anti-collagen IVα1 antibody (Abcam, Cambridge, UK) and anti-β-actin antibody (CST, Boston, USA).

### 2.4 RNA Interference

siRNA duplexes (100 nM) with Lipofectamine RNAiMAX (Invitrogen) were used to knock down COL4A1 in cell lines according to the manufacturer’s instructions. List of siRNA sequences:

COL4A1-1 F: 5′- AGGCGAAAUUGGAGAGAAATT -3′, R: 5′- UUUCUCUCCAAUUUCGCCUTT-3′, COL4A1-2: F: 5′- CGGAAAAGAUGGUGACAAATT -3′, R: 5′- UUUGUCACCAUCUUUUCCGTT-3′ NC: UUCUCCGAACGUGUCACGUTT

### 2.5 Cell Proliferation Assay and Colony Formation Assay

The Cell Counting Kit-8 (CCK-8, Biomiky, Japan) assay was used to determine cell proliferation. Cells were seeded into 96-well plates at a density of 8×103 cells/well overnight. Then, the cells in each well were stained with 10 μL CCK-8 and incubated for 2 h at 37°C. Cell viability was measured according to the different absorptions at wavelengths of 450 nm using a spectrophotometer (BMG Labtech, Aylesbury, UK). For the colony formation assay, cells were transfected with the indicated siRNAs and plated into 6-well plates at 200-400 cells/well. The culture supernatant was changed every three days until visible colonies formed. Then, colonies were fixed with 4% paraformaldehyde and stained with 0.1% crystal violet for counting.

### 2.6 Cell Invasion and Migration Assay

Transwell chambers (Corning, Shanghai, China) were used to determine cell invasion according to the instructions provided by the manufacturer. Medium with 10% FBS was added to the bottom chamber, and 1×105 infected cells in serum-free medium were seeded into the upper chamber and incubated for 48 hours. Then, invasive cells were fixed with 4% paraformaldehyde and stained with 0.1% crystal violet for cell counting. For the migration assay, cells were seeded into 6-well plates. A 250 μL pipette tip was used to scratch perpendicular to the culture plate. After scratching, the cells were rinsed gently with PBS to remove the cells scratched by the pipette tip so as not to affect the subsequent shooting. The time is set to 0 h to take a picture. After adding the medium, we took a picture again to observe the cell migration after incubation for 24 hours. ImageJ software was used to measure the scratch distance.

### 2.7 Tissue Microarray and Immunohistochemistry

GC tissue microarrays (No: HLugA180Su08) accompanied by pathological features, including tumor size, histological grade, lymph node stage and metastasis, and survival, were obtained from Shanghai Xinchao Company and approved by the National Human Genetic Resources Sharing Service Platform. The tissue microarray sections were deparaffinized in xylene and then rehydrated in a graded series of ethanol from 100% to 75%. The endogenous enzyme of the samples was deactivated in 3% hydrogen peroxide for 15 minutes and then flushed with water for 2-3 minutes. A 10 mM EDTA buffer with pH 8 was used for antigen retrieval. Slides are placed into the pressure cooker along with the retrieval solution and heated until the solution reaches boiling point, where it is maintained for 1-4 minutes. Samples were blocked in 1% peroxide solution for 10 min, followed by washing 3 times with TBST. Slides were blocked in 3% bovine serum albumin, incubated with anti-collagen IV (1:1000) overnight at 4°C, incubated in HRP-conjugated secondary antibody (1:3000) for 1 h at 25°C and then visualized with 3,3′-diaminobenzidine (DAB) for 10 min. Nuclei were stained blue by hematoxylin counterstaining. Images were obtained randomly from histological fields in each section at 400× magnification using an Olympus BX63 microscope (Olympus, Japan). The images were quantified by ImageJ software (National Institutes of Health, USA) to determine the percentages of collagen IV-positive cells. Two pathologists performed the assessment independently. Staining intensity score criteria were as follows: absent, weak, moderate, or strong was scored as 0 (negative), 1 (1+), 2 (2+), or 3 (3+), respectively. The score of positive staining percentage was as follows: 0 (absent), 1% to <25% positive staining was scored as 1 (focal), ≥25% to <50% positive staining was scored as 2 (diffuse), ≥50% to <75% positive staining was scored as 3 (diffuse) or ≥75% positive staining was scored as 4 (diffuse). The final score was calculated by multiplying the staining intensity score by the percentage of positive staining score. The median was taken as the cutoff value and the total score ≤3 was divided into the low expression group, and the total score >3 was divided into the high expression group.

### 2.8 Data Acquisition

The RNA-sequencing (RNA-seq) data of the GC cohort with clinicopathological information were downloaded and extracted from the TCGA (https://tcga-data.nci.nih.gov/) database.

### 2.9 Survival Analysis

For survival analysis, patients were divided into high-expression and low-expression groups according to COL4A1 mRNA expression in TCGA using a cut-off value defined by optimal cut-off value. The survival rates of the two groups were compared by log-rank test using the Kaplan-Meier Plotter online database (www.kmplot.com) [28].

### 2.10 Identification of Differentially Expressed Genes (Degs)

The DEGs were identified by the “limma” package of R v3.5.0 software comparing gastric carcinoma tissues with normal controls [29]. For continuous data, the Student’s *t-*test was used to compare the differences between the two groups. *p* < 0.05, and cut-off values were set as a fold change >2.0.

### 2.11 Clinicopathological Analysis

The relationship between COL4A1 mRNA expression and clinicopathological features was analysed using corrected chi-square, Wilcoxon rank-sum, and Kruskal-Wallis rank-sum tests from the R v3.5.0 software package.

### 2.12 Enrichment Analysis of The Degs

The RNA-seq data were obtained from the TCGA dataset. Gene Ontology (GO), Kyoto Encyclopedia of Genes and Genomes (KEGG) pathway, and Hallmark enrichment analyses were performed using the R v3.5.0 software package to understand the genes that were positively and negatively associated with COL4A1. The GO analysis used here included biological process (BP), cellular composition (CC), and molecular function (MF). We used the R software package “ClusterProfilter” for enrichment analysis. In multiple GSEA plot analyses, the MSigDB category (h.all.v7.5.1.symbols.gmt and c2.cp.kegg.v7.4.symbols.gmt) was set as the reference gene set, which was achieved with the R packages “enrichplot” and “clusterProfiler”. The top 5 terms are presented here. Statistical significance was determined by a *p* value <0.05 and a q-value < 0.25.

### 2.13 Immunological Features of Col4a1

The stromal, immune, and ESTIMATE scores were estimated by “ESTIMATE” software from the R package [30], and the scores were compared between the high and low COL4A1 expression groups. To explore the relationship between COL4A1 and GC microenvironment immune cells, the CIBERSORT algorithm was used to detect differential immune cell types in the high and low COL4A1 expression groups [31]. The Pearson coefficient assessed significant differences between the two subgroups.

### 2.14 Drug Sensitivity Analysis

The data of the half-maximal inhibitory concentration (IC_50_) of drugs in differentially expressed genes were acquired from the Genomics of Drug Sensitivity in Cancer (GDSC) project (https://www.cancerrxgene.org/). The relationship between drug IC50 and CLO4A1 expression was estimated with the pRRophetic package [32].

### 2.15 Nomogram Construction

The nomogram was constructed based on 8 parameters using RMS (6.2-0, https://cran.rproject.org/web/packages/rms/index.html) and SURVIVAL (3.3-1, https://cran.r-project.org/web/packages/survival/index.html) after recalculating 200 times using the bootstrap method. We calculated a nomogram score to predict the probability of 1-, 3-, and 5-year OS (overall survival). Assessment of the calibration capability of the nomogram was performed by plotting calibration curves.

### 2.16 Statistical Analysis

COL4A1 expression in GC tissues and normal control tissues was analysed by the Wilcoxon rank-sum test. Kaplan-Meier curves and log-rank tests were used to compare the OS, PFS, and PPS between high and low COL4A1 expression. In addition, ROC analysis and the frequently used method for binary assessment were conducted using the pROC package (1.17.0.1) to assess the diagnostic capability. The computed AUC value from 0.5 to 1 indicates the discriminative potential from 50% to 100%. In all tests, a *p*-value < 0.05 was considered statistically significant (Supplementary Figs. **1**-**3**).

## 3 RESULTS

### 3.1 Clinical Characteristics of Col4a1 in Gc from Tcga Datasets

**Fig. (1):**
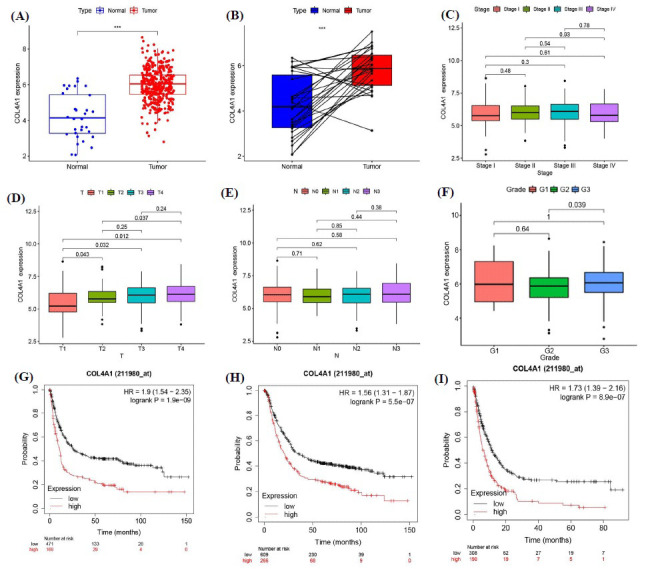
Clinical characteristics of COL4A1 in GC from TCGA datasets. (**A**) The expression level of COL4A1 between 375 GC patients and 32 normal control tissues obtained from database. (**B**) COL4A1 expression levels were compared for 27 paired tumor and adjacent normal tissue from the TCGA database. (**C**) Correlation between the expression of COL4A1 and the pathologic stage of GC. (**D**) Correlation between the expression of COL4A1 and the T stage of GC. (**E**) Correlation between the expression of COL4A1 and the N stage of GC. (**F**) Correlation between the expression of COL4A1 and the histologic grade of GC. Kaplan-Meier survival curve analysis for (**G**) Overall survival, (**H**) Progression free survival and (**I**) Post progression survival of GC patients based on the COL4A1 expression. *p < 0.05, **p < 0.01, ***p < 0.001.

Transcriptome sequencing data of 375 GC patients and 32 normal controls were obtained from the TCGA database. COL4A1 was statistically upregulated in GC compared to normal tissue and was almost exclusively increased in tumors when analysed in paired cases (Figs. **1A** and **B**). To investigate the relationship between COL4A1 and other prognostic parameters of GC, the clinical characteristics of patients, such as age, sex, grade, stage, tumor invasion (T), lymph node metastasis (N), and distant metastasis (M), were extracted from the TCGA database, and correlation analysis with COL4A1 was carried out (Figs. **1C**-**F**, Fig. **S1A**-**C**). Compared to T1, gastric cancer (GC) with T2-4 stages showed a stronger association with COL4A1 overexpression (Fig. **1D**). COL4A1 expression was significantly higher in grade 3 than in grade 2, although no significant difference was observed between grade 1 and grades 2 or 3, likely due to limited data (Fig. **1F**). However, no correlation was found between COL4A1 expression and stage, N, M, age, or sex (Figs. **1C**, **E**, Figs. **S1A**-**C**). These results showed that patients with Grade 3 and T2-4 GC were more predisposed to COL4A1 overexpression. Survival curves were calculated based on the Kaplan‒Meier Plotter online database, showing dramatically inferior overall survival (OS), progression-free survival (PFS) and post-progression survival (PPS) with COL4A1 overexpression (Figs. **1G**-**I**). The above results suggested that COL4A1 was upregulated in GC, indicating a poor prognosis.

### 3.2 Verification of the Prognostic Significance of Col4a1 in Gc Tissues

**Fig. (2):**
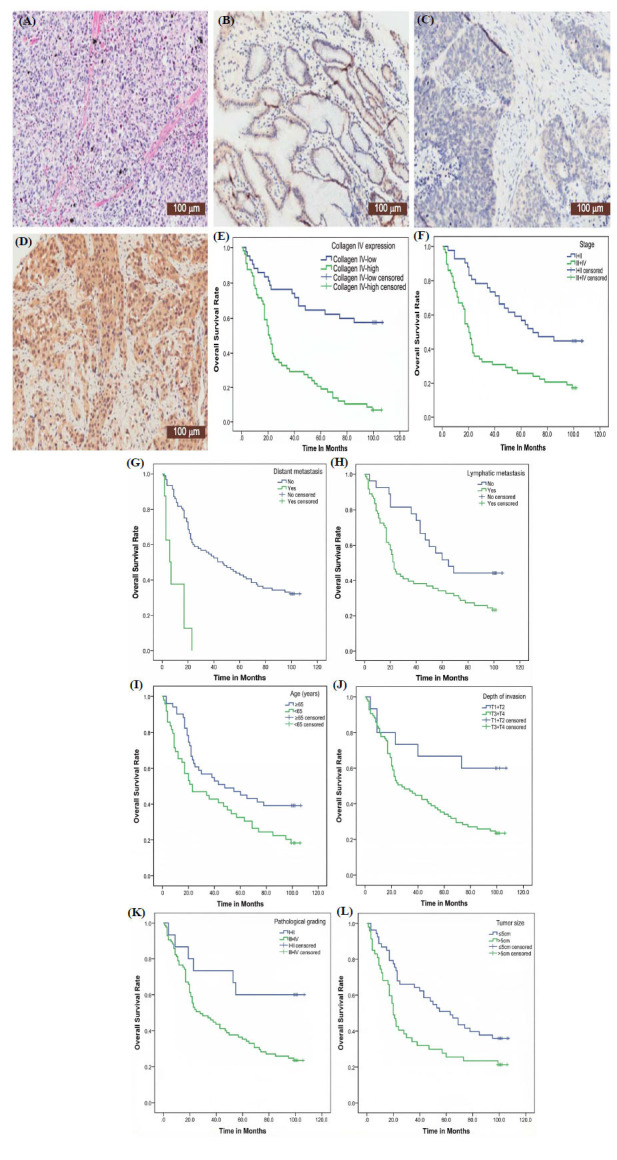
Verification of prognostic significance of COL4A1 in GC tissues. (**A**) HE staining of collagen IV protein was detected in GC tissues. (**B**) Collagen IV was negatively expressed in normal tissues. (**C**) Collagen IV was weakly expressed in GC tissues. (**D**) Collagen IV was strongly expressed in GC tissues. Univariate analysis using Kaplan-Meier method (**E**-**L**). Kaplan-Meier curves based on collagen IV (**E**), stage (**F**), distant metastasis (**G**), lymphatic metastasis (**H**), age (**I**), invasion (**J**), pathological grade (**K**) and tumor size (**L**) .

GC tissue microarrays (No: HLugA180Su08) harboring clinicopathological characteristics, including tumor size, histological grade, lymph node stage and metastasis, and survival, were used to verify the findings from the TCGA database. COL4A1 expression was defined by immunohistochemical analysis, and COL4A1 was upregulated in GC compared to normal tissues (Figs. **2A**-**D**). We investigated the correlation between collagen IV expression and clinical pathological characteristics in 100 GC patients. Tumor invasion, lymph node metastasis, and stage were positively correlated with COL4A1 expression (Table **1**). This result verified the relationship between COL4A1 and tumor invasion in the TCGA database. Moreover, the univariate analysis indicated that COL4A1 expression, age, pathological grade, tumor size, invasion, lymphatic metastasis, distant metastasis, and stage were obviously correlated with OS, and survival curves were generated using the Kaplan‒Meier method (Table **2**, Figs. **2E**-**L**). Multivariate analysis using the Cox proportional hazards model indicated that high COL4A1 expression, tumor size>5 cm, and distant metastasis were independent prognostic factors for OS in patients with GC (Table **3**). Based on those parameters, these data supported the effectiveness of the nomogram constructed.

### 3.3 Identification of Col4a1 Co-expression Genes and Enrichment Analysis

**Fig. (3):**
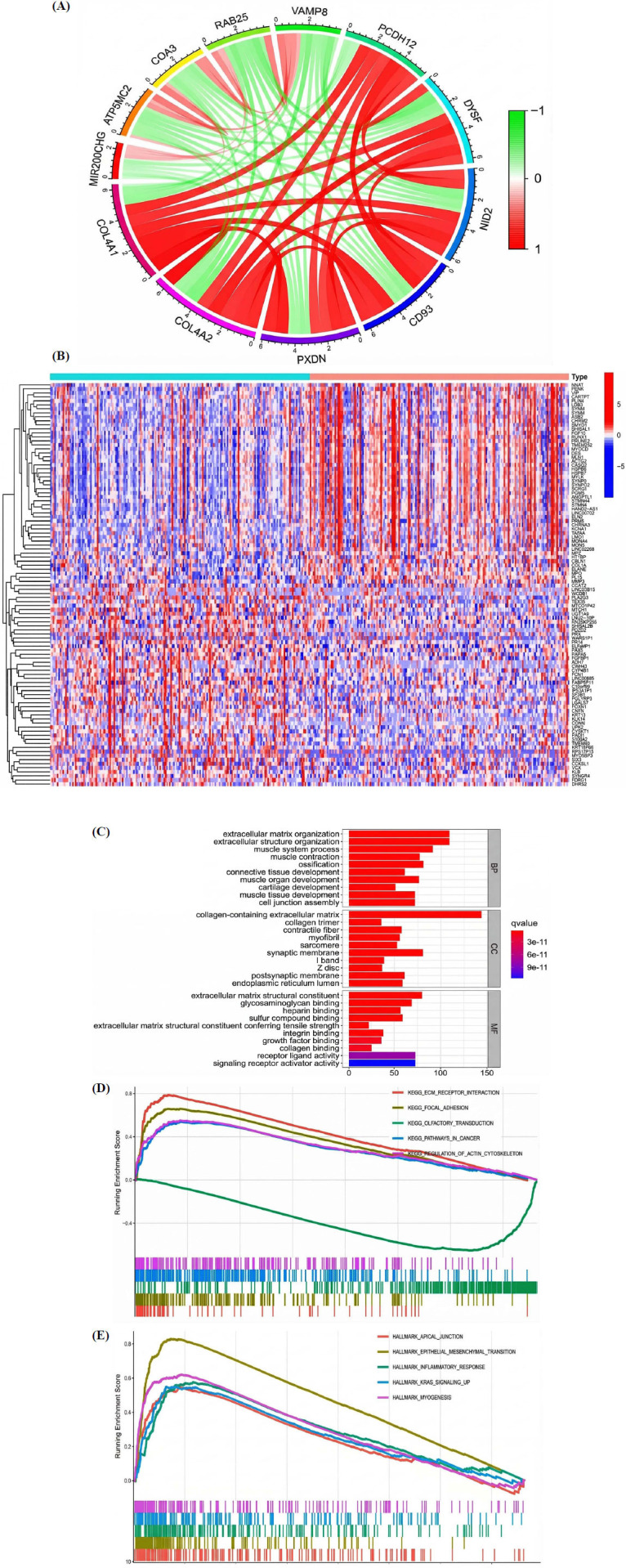
Identification of COL4A1 co-expression Genes and enrichment analysis.(**A**) Co-expression gene related of COL4A1. (**B**) Heatmap of differential genes related of COL4A1. (**C**) GO terms enrichment analysis of COL4A1 co-expression genes. (**D**) KEGG pathway enrichment analysis of COL4A1 co-expression genes. (**E**) Hallmark enrichment analysis of COL4A1 co- expression genes.

To better investigate the mechanism of COL4A1 in GC, we screened out the genes co-expressed with COL4A1. The co-expression circle map revealed that COL4A1 was positively regulated by PCDH12, DYSF, NID2, CD93, and PXDN, while being negatively regulated by VAMP8, RAB25, COA3, and ATP5MC2 (Fig. **3A**). These findings were also confirmed through differential expression analysis but were not included in the heatmap, as the expression levels of these genes did not rank among the top 50 (Fig. **3B**). Carcinogenic genes such as PLIN4, HSBP6/7, FGF10, and TMEM252 were upregulated in the COL4A1 overexpression group, whereas the longevity-related gene KRT15 and the anticancer gene DHRS2 were associated with low COL4A1 expression. GO analysis using the “Cluster Profiler” package showed that COL4A1 was mainly related to extracellular matrix organization in the BP category, collagen-containing extracellular matrix in the CC category, and extracellular matrix structural constituent in the MF category (Fig. **3C**). KEGG analysis showed that COL4A1 was mainly involved in an extracellular matrix formation, adhesion, and cancer pathways (Fig. **3D**). Hallmark analysis showed that COL4A1 was related to apical junctions, epithelial-mesenchymal transition (EMT), inflammation, KRAS signaling and myogenesis (Fig. **3E**). These results suggested that COL4A1 promotes GC progression by multiple pathways, including cancer cell pathways and tumor microenvironment (TME).

### 3.4 Col4a1 Knockdown Suppressed the Invasion and Migration of Gc Cells

**Fig. (4):**
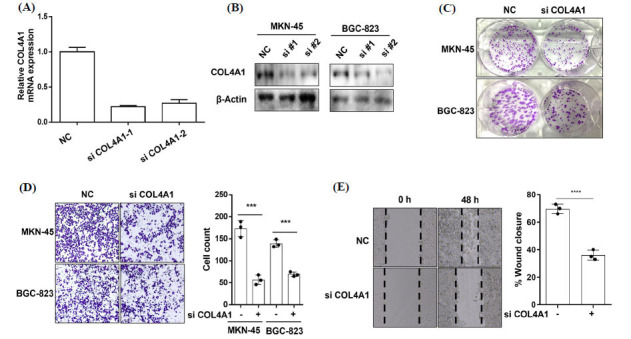
COL4A1 knockdown suppressed the invasion and migration of GC cells.(**A**) COL4A1 siRNA was transfected into MKN-45 cells by lipofectamine RNAi MAX for 48 h, and then COL4A1 gene expression was analyzed by Q-PCR. (**B**) COL4A1 siRNA was transfected for 48 h followed by Western-blot analysis. (**C**) Clone formation was analyzed after COL4A1 siRNA transfection. (**D**) Cells invasion was analyzed by transwell chambers after COL4A1 siRNA transfection. (**E**) Scratch test was analyzed after COL4A1 siRNA transfection. *p < 0.05, **p < 0.01, ***p < 0.001.

To investigate the biological functions of COL4A1 in cancer cells, siRNAs were transfected into two gastric cell lines, MKN-45 and BGC-823. COL4A1 siRNA was proven by mRNA and protein expression (Figs. **4A**, **B**). Unexpectedly, the proliferation of GC cells was unregulated by COL4A1 (Figs. **S2A**, **B**). After siRNA-COL4A1 transfection, cell tumorigenicity, and expansion capacity were reduced visibly by colony formation assay (Fig. **4C**). TCGA data confirmed that COL4A1 plays an important role in regulating extracellular matrix organization. Therefore, the effect of COL4A1 expression on the invasion and migration of GC cells was evaluated using Transwell chambers and scratch tests. In the siRNA-COL4A1 group, fewer cells in the Transwell chambers passed through the bottom membrane compared with those in the control group (Fig. **4D**). In the scratch test, cell migration was inhibited by COL4A1 knockdown (Fig. **4E**). These results demonstrated that COL4A1 knockdown inhibited the tumorigenicity, invasion, and migration of GC cells.

### 3.5 Immunological Features of Col4a1 in the TME of Gc

**Fig. (5):**
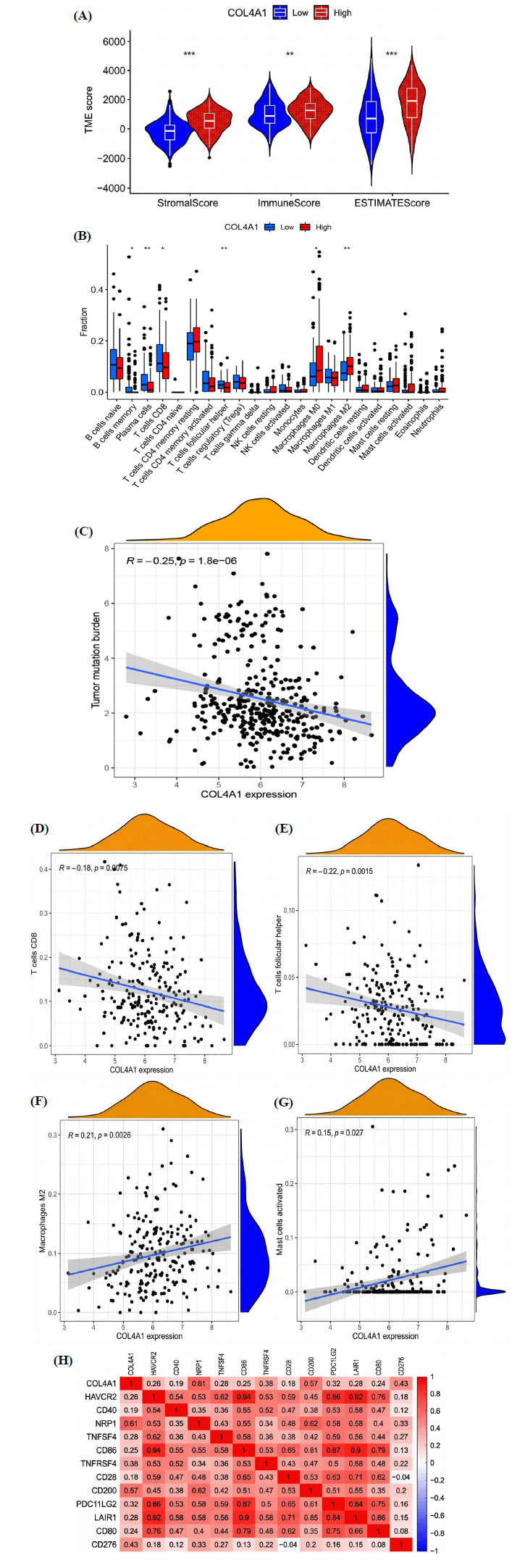
Immunological features of COL4A1 in the TME of GC.(**A**) TME score in stromal, immune and estimate zone between high and low COL4A1 expression was calculated by “ESTIMATE” software. (**B**) immune-related cells between high and low COL4A1 expression were distinguished by “ESTIMATE” software. The relationship between COL4A1 and tumor mutation burden (**C**), CD8^+^ T cells (**D**), follicular helper T cells (**E**), macrophages M2 (**F**) and activated mast cells (**G**). (**H**) Heatmap of differential immune checkpoint genes related of COL4A1.

COL4A1 plays an important role in the TME of GC. Using “ESTIMATE” software, we found that COL4A1 overexpression was accompanied by higher stromal scores and immune scores, which indicate abundant stromal and immune cell infiltration (Fig. **5A**). The immune-related cells were analysed by multiple algorithms, which indicated that TMB, CD8^+^ T cells and follicular helper T cells were obviously negatively correlated with COL4A1 expression (Figs. **5B**-**E**). These immune cells were demonstrated to have antitumor immunity. However, M2 macrophages and mast cells that suppressed antitumor immunity were positively correlated with COL4A1 (Figs. **5F**, **G**). In addition, immune checkpoint genes were investigated showing that COL4A1 was positively correlated with NRP1, CD200 and CD276, which were reported to impair antitumor immunity (Fig. **5H**) [33-35]. The above results suggested that COL4A1 promotes GC by remodelling the immune microenvironment.

### 3.6 Col4a1 Overexpression Reduced Drug Sensitivity

**Fig. (6):**
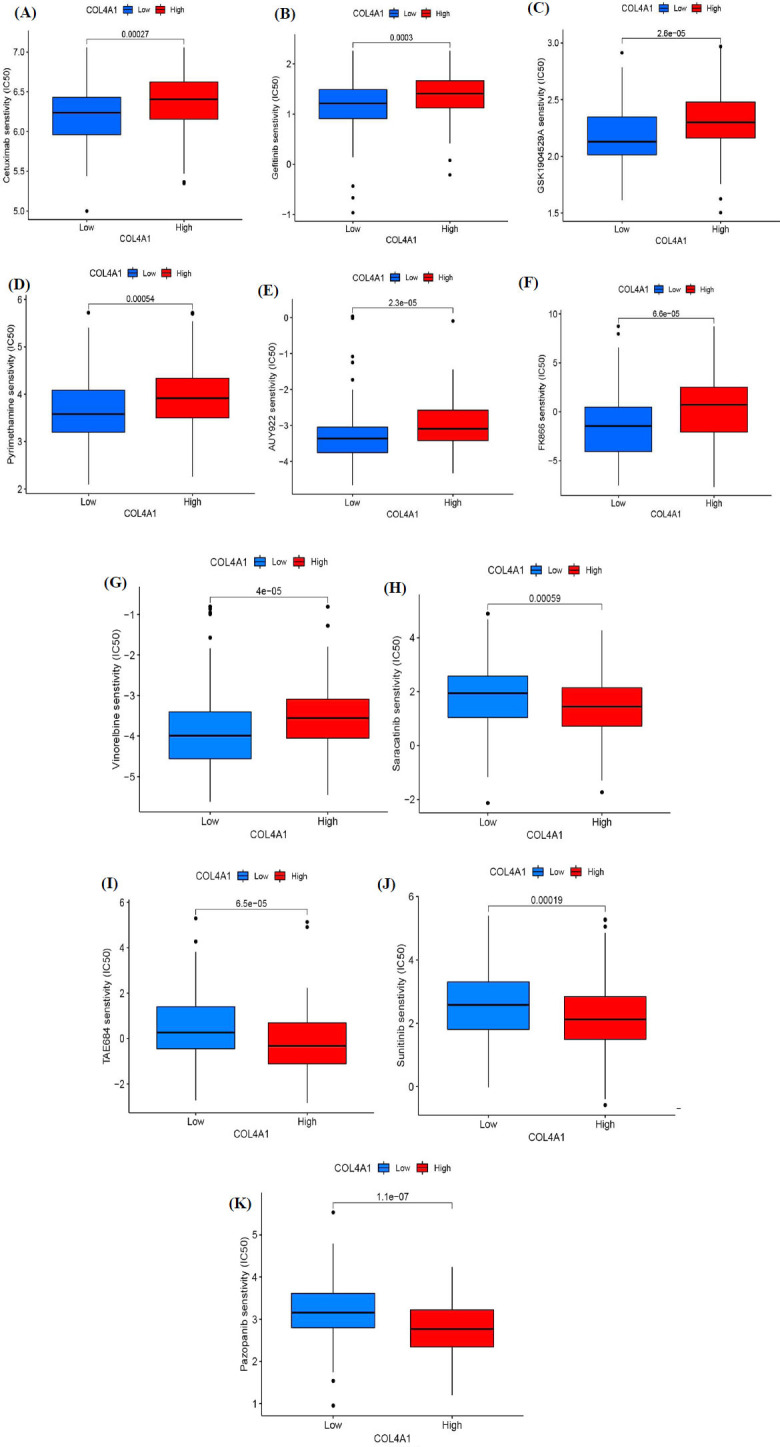
COL4A1 overexpression reduced drug sensitivity. The relationship between COL4A1 and drug sensitivity. Cetuximab (**A**), gefitinib (**B**), GSK1904529A (**C**), pyrimethamine (**D**), AUY922 (**E**), FK866 (**F**), vinorelbine (**G**), saracatinib (**H**), TAE684 (**I**), sunitinib (**J**) and pazopanib (**K**).

To better understand the correlation between COL4A1 differential expression and therapeutic strategy, drug sensitivity was predicted by the R software package “pRRophetic” from the GDSC database. The top 23 drugs with the highest differences showed that COL4A1 overexpression displayed multidrug resistance, including inhibitors targeting signaling pathways such as EGFR, IGFR, STAT3, JNK, PDK-1, MARK and HRAS and other potential antitumor molecule inhibitors such as HSP90, NAMPT, HDAC, MDM4 and PARP (Figs. **6A**-**F**, **S3A**-**H**). In addition, patients with COL4A1 overexpression did not benefit from chemotherapy, such as vinorelbine, which in turn deteriorated survival (Fig. **6G**). However, inhibitors targeting SRC, ALK and PI3K and some multitarget inhibitors, such as sunitinib and pazopanib, had the advantage in COL4A1 overexpression samples, suggesting an optimal scheme (Figs. **6H**-**K**, **S3I**-**L**).

### 3.7 A Reliable Nomogram Based on Col4a1 was Established for the Prognostic Prediction of Gc

**Fig. (7):**
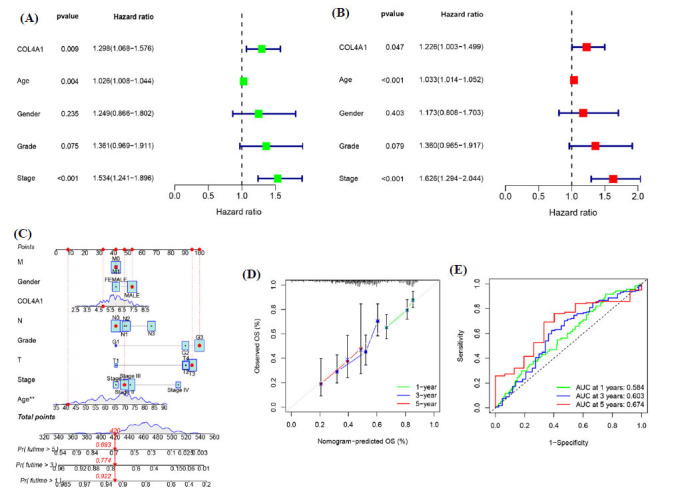
A reliable nomogram based on COL4A1 was established for prognostic prediction of GC. Risk factors for GC survival were analyzed by univariate cox regression (**A**) and multivariate cox regression (**B**). (**C**) Nomogram survival prediction constructed by metastasis, gender, COL4A1 expression, lymph node metastasis, grade, tumor invasion, stage and age for GC patients to predict 1-, 3-, and 5-year survival possibility. (**D**) The accuracy and specificity (**E**) of the nomogram was verified by The ROC analysis.

Based on the TCGA cohort, univariate and multivariate analyses showed that COL4A1 and stage were statistically significant in predicting OS in GC patients (Fig. **7A**, **B**). To provide a quantitative prediction method, a nomogram model was developed based on COL4A1 to predict survival using 8 parameters, including distant metastasis, sex, COL4A1 expression, lymphatic metastasis, grade, tumor invasion, stage and age (Fig. **7C**). The projected OS rates of patients with GC at 1, 3, and 5 years were calculated, and the calibration curve confirmed an acceptable level of accuracy for close to the ideal curve (Fig. **7D**). The ROC analysis indicated that the nomogram model could accurately distinguish patients with GC who would achieve better clinical outcomes (area under the curve AUC of 1-year survival, 0.584; AUC of 3-year survival, 0.603; AUC of 5-year survival, 0.674) (Fig. **7E**).

## 4 DISCUSSION

Despite significant progress in immunotherapy for unresectable and/or metastatic GC (particularly anti-PD-1 combined with anti-CTLA-4), the objective response rate is between 27-60%, with median PFS for approximately 7 months [10, 36]. Anti-claudin 18.2 combined with chemotherapy acquired an objective response rate of 73.3% in patients with CLDN18.2-positive GC reported at the 2023 ASCO Annual Meeting. However, this groundbreaking discovery only benefited a small number of people, with 38.4% CLDN18.2 positive rate [37, 38]. The success of anti-CLDN18.2 suggests that the development of new therapeutic markers will still be the direction of GC in the next five years. Our study demonstrates a new paradigm of potential therapeutic target that not only determines the malignant phenotype of tumor cells but also regulates the tumor matrix and TME, which may lead the direction of the next breakthrough target for GC.

Previous studies have indicated that COL4A1 is positively correlated with poor prognosis by analysing the GEO database, and the main reason is to promote the proliferation and invasion of tumor cell lines. However, the above conclusions need to be verified in GC tissues. COL4A1, encoding the α subunit of collagen IV, is a member of the extracellular matrix that determines tumor cell migration and TME more than tumor itself. Therefore, further research is needed to explore the role of COL4A1 in the TME. This study investigated RNA-seq information in the TCGA database and found that COL4A1 was also upregulated in GC, especially in patients with grade 3 and T2-4 disease. To validate this hypothesis, GC tissue microarray immunohistochemical analysis also confirmed that tumor invasion, lymph node metastasis and stage were positively correlated with COL4A1 expression. The above results established the prognostic value of COL4A1 in GC patients. COL4A1 overexpression in GC was mainly related to extracellular matrix construction and organization and subsequently promoted tumor adhesion and migration by GO and KEGG analysis. Moreover, the positive correlation between COL4A1 and KRAS signaling might result in malignant proliferation of tumor cells. Interestingly, COL4A1 knockdown in the gastric cell line blocked the migration and invasion of GC but not cell proliferation. Unlike the CCK-8 assay, the colony formation assay showed enhanced tumorigenicity due to enhanced KRAS signaling by COL4A1 overexpression, which should be noted. The extracellular matrix (ECM) usually constructed by tumor plays an important role in the immunosuppressive microenvironment by increasing density to hamper immune cell migration into tumor islets and inducing immunosuppressive cell remodelling, thus resulting in the inefficacy of immunotherapies [39]. This study analysed the correlation of COL4A1 expression and immune-related cells. It showed that TMB, CD8^+^ T cells, and follicular helper T cells were obviously negatively correlated with COL4A1 expression. In contrast, M2 macrophages, mast cells and checkpoint genes such as NRP1, CD200 and CD276, which were reported to suppress antitumor immunity, were positively correlated with COL4A1. Based on the above results, COL4A1 promoted GC progression by regulating both tumor cells and TMB, which explained the reason that COL4A1 overexpression induced multidrug resistance by drug sensitivity analysis, and also indicated that some inhibitors targeting SRC, ALK, and PI3K or multitarget inhibitors might be strategies for patients with COL4A1 overexpression.

Finally, the clinical application of COL4A1 was reflected in a nomogram model that was developed based on 8 parameters, including COL4A1 expression, which makes it convenient to predict the prognosis of GC patients. Collectively, this study emphasized COL4A1 as a prognostic biomarker and a potential therapeutic marker for GC.

The limitations of this work are obvious. First, COL4A1 overexpression is involved in the malignant phenotype of tumor. Bioinformatics analysis found that COL4A1 overexpression may be positively correlated with KRAS signaling. However, this study was underpowered to verify this biological mechanism of COL4A1 *in vitro* and *in vivo*, especially the regulation of KRAS signaling. Furthermore, although the negative correlation of COL4A1 with antitumor immunity was revealed in this article, it was not validated *in vivo*. To confirm the value of this new therapeutic target, its anti-tumor effect and regulation of the anti-tumor immune microenvironment need to be further verified in COL4A1 knockout mice in the future.

## CONCLUSION

COL4A1 is identified as a prognostic marker and potential therapeutic target in gastric cancer. Its overexpression correlates with poor prognosis, tumor invasion, and immunosuppression. A nomogram based on COL4A1 can predict patient outcomes. Future research should validate these findings and explore targeted therapies.

## Figures and Tables

**Table 1:** Correlation between collagen IV expression and clinical pathological characteristics in 100 gastric cancer patients.

**Clinical Characteristics**	**n=100**	**COL4A1 Expression**		***x^2^***	***P* Value **
		**Low**	**High**		
Gender					
Female	36	14	22	0.388	0.533
Male	64	29	35		
Age (years)					
≤65	51	26	25	2.704	0.100
>65	49	17	32		
Pathological grading					
I+II	15	8	7	0.769	0.381
III+IV	85	35	50		
Tumor size					
≤5 cm	53	25	28	0.800	0.371
>5 cm	47	18	29		
Depth of invasion					
T1+T2	15	10	5	4.033	0.045
T3+T4	85	33	52		
Lymphatic metastasis					
Yes	73	26	47	6.014	0.014
No	27	17	10		
Distant metastasis					
Yes	8	2	6	0.490	0.484
No	92	41	51		
Stage					
I/II	42	24	18	5.910	0.015
III/IV	58	19	39		

**Table 2:** Univariate analysis in gastric cancer by Collagen IV expression and clinicopathological characteristics.

**Variable**	**Overall Survival Time**		
	**95% CI**	***x^2^***	***P* Value**
COL4A1 expression			
Low	62.983-87.296	29.642	0.000
High	25.752-41.792		
Gender			
Female	36.408-61.981	0.294	0.588
Male	42.470-62.811		
Age (years)			
≤65	48.315-70.822	5.847	0.016
>65	32.211-54.034		
Pathological grading			
I+II	54.268-95.866	5.731	0.017
III+IV	38.894-55.553		
Tumor size			
≤5 cm	51.060-72.411	6.052	0.014
>5 cm	28.794-51.121		
Depth of invasion			
T1+T2	53.302-96.165	5.581	0.018
T3+T4	38.994-55.571		
Lymphatic metastasis			
Yes	35.098-52.875	5.743	0.017
No	55.097-82.977		
Distant metastasis			
Yes	4.172-15.328	26.889	0.000
No	46.927-63.551		
Stage			
I/II	58.696-81.352	14.075	0.000
III/IV	27.974-46.819		

**Table 3:** Multivariate analysis in gastric cancer by Collagen IV expression and clinicopathological characteristics.

**Variable**	**Overall Survival Time**		
	**HR**	**95% CI**	***P* Value**
COL4A1 (high *vs.* low)	3.213	1.809-5.706	0.000
Gender (male *vs.* female)	0.689	0.416-1.141	0.147
Age (≤65 *vs.* >65)	1.581	0.971-2.572	0.065
Pathological grading(III+IV *vs.* I+II)	1.802	0.739-4.396	0.195
Tumor size (≤5 cm *vs.* >5 cm)	1.684	1.028-2.757	0.038
Invasion depth (T3+T4 *vs.* T1+T2)	1.460	0.577-3.692	0.424
Lymphatic metastasis (Yes *vs.* No)	1.073	0.434-2.652	0.879
Distant metastasis(Yes *vs.* No)	4.378	1.911-10.030	0.000
Stage (III+IV *vs.* I+II)	1.689	0.732-3.900	0.219

## Data Availability

The data and supportive information are available within the article.

## LIST OF ABBREVIATIONS

COL4A1: Collagen Type IV Alpha 1 Chain
EBV: Epstein‒Barr Virus
TMB: Tumor Mutation Burden
MSI-H: MSI High
TCGA: The Cancer Genome Atlas
GC: Gastric Cancer
TME: Tumor Microenvironment
