# Supplementary material for: COL4A1 Promotes Gastric Cancer Progression by Regulating Tumor Invasion, Tumor Microenvironment and Drug Sensitivity
Source: Curr Med Chem. 2025 Apr 7;33(11):2190–210. doi: 10.2174/0109298673351943250314074632 (PMC13478938; doi:10.2174/0109298673351943250314074632)
Supplement: Supplementary file 1 [file CMC-33-11-2190_SD1.pdf]

Supplementary Material

COL4A1 Promotes Gastric Cancer Progression by Regulating Tumor Invasion, Tumor Microenvironment and Drug Sensitivity

Xiaojun Qian[1,#], Wei Jia[1,#], Yuntian Li[2], Jian Chen[1], Jinguo Zhang[1,*] and Yubei Sun[1,*]

[1]*Department of Medical Oncology, The First Affiliated Hospital of USTC, Division of Life Sciences and Medicine, University of Science and Technology of China, Hefei, 230001, P.R. China;* [2]*Department of Radiation Oncology, The First Affiliated Hospital of USTC, Division of Life Sciences and Medicine, University of Science and Technology of China, Hefei, 230001, P.R. China*

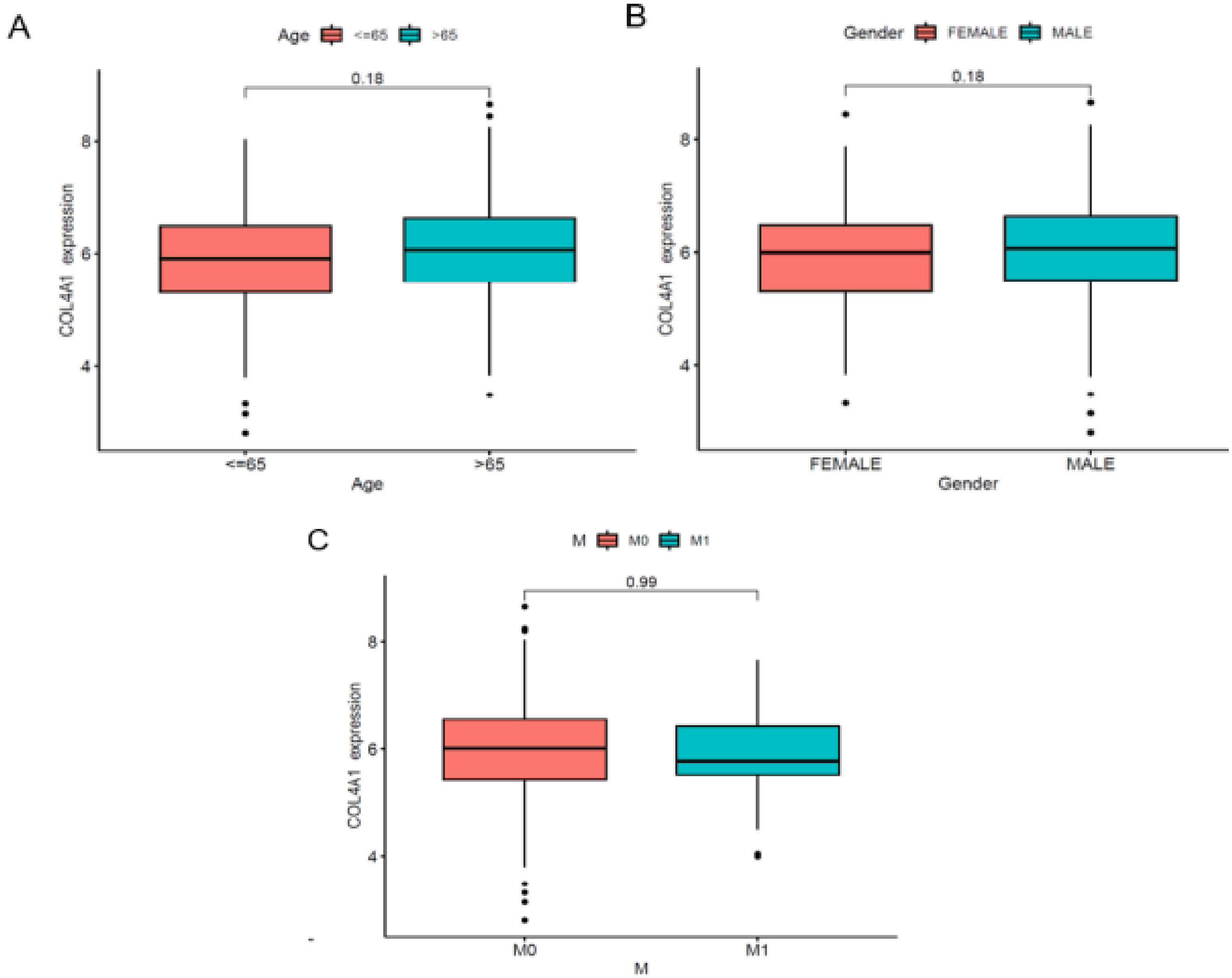

Fig. (S1). Clinical characteristics that not associated with COL4A1. (**A**) Correlation between the expression of COL4A1 and age of GC patients. (**B**) Correlation between the expression of COL4A1 and gender of GC patients. (**C**) Correlation between the expression of COL4A1 and metastasis of GC.

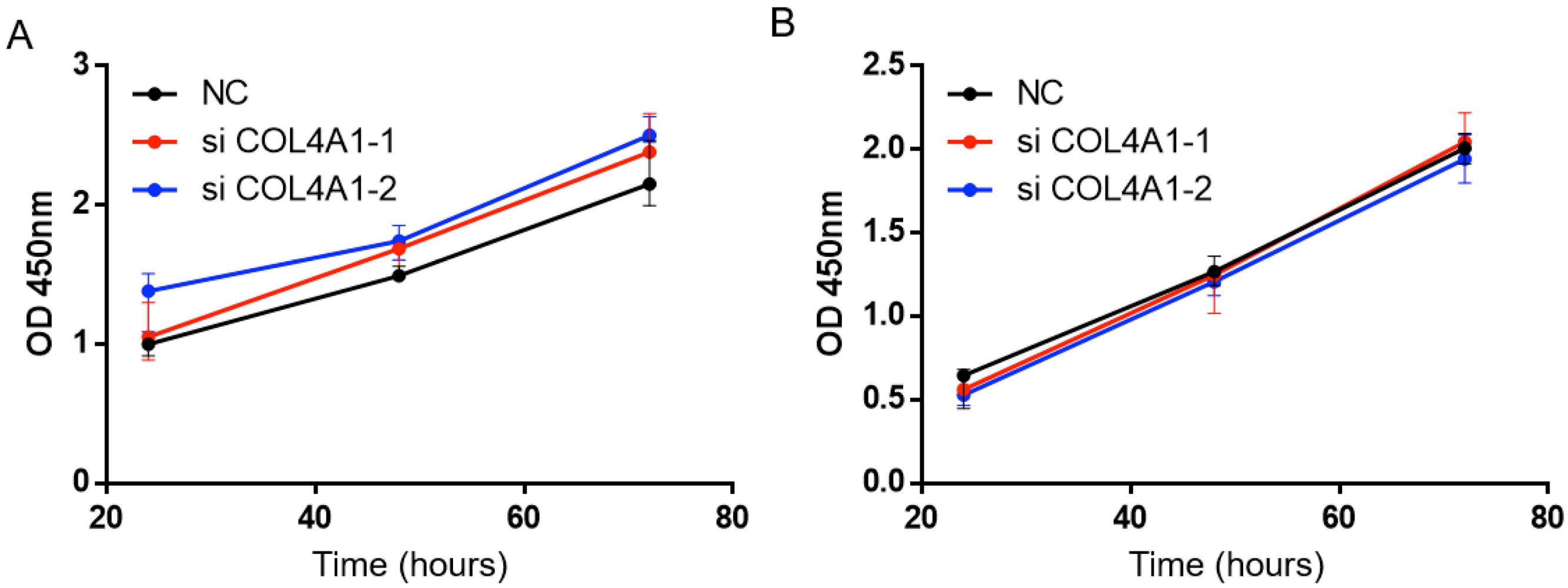

Fig. (S2). COL4A1 knockdown had no effect on the proliferation of GC cells. Cell proliferation was measured by CCK-8 after COL4A1 knockdown in MKN-45 (**A**) and BGC-823 (**B**).

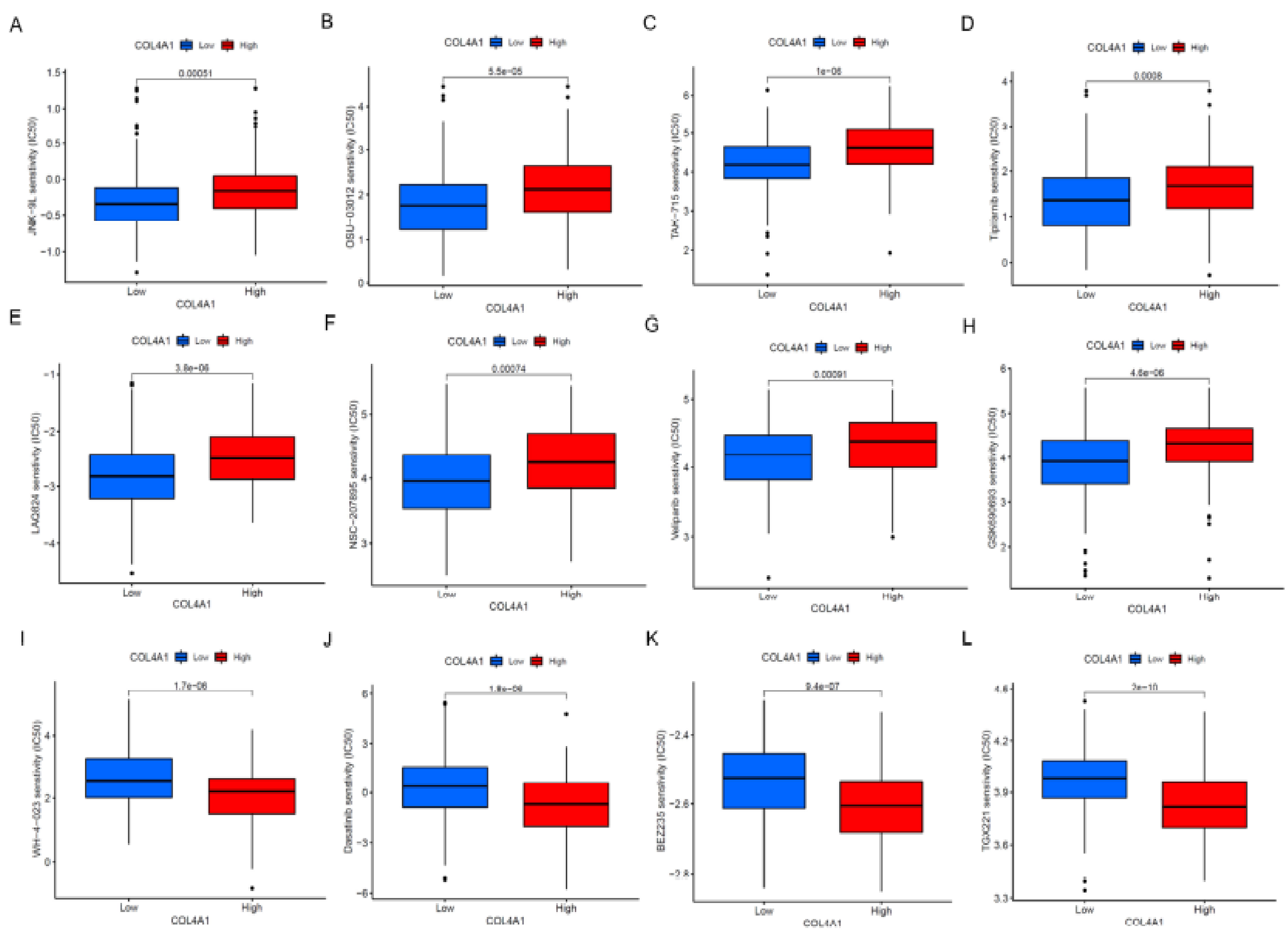

Fig. (S3). The relationship between COL4A1 and drug sensitivity. JNK-9L (**A**), OSU-03012 (**B**), TAK-715 (**C**), tipifarnib (**D**), NVP-LAQ824 **(E)**, NSC-207895 (**F**), veliparib (**G**), GSK690693 (**H**), WH-4-023 (**I**), dasatinib (**J**), BEZ-235 (**K**) and TGX-221 (**L**).
